# Optimal surgical approaches for esophageal epiphrenic diverticulum: literature review and our experience

**DOI:** 10.1007/s12328-023-01765-2

**Published:** 2023-02-01

**Authors:** Yuta Sato, Yoshihiro Tanaka, Shinya Ohno, Masahide Endo, Naoki Okumura, Takao Takahashi, Nobuhisa Matsuhashi

**Affiliations:** Department of Gastroenterological Surgery and Pediatric Surgery, Gifu Graduate School of Medicine, 1-1 Yanagido, Gifu City, Gifu Prefecture 501-1194 Japan

**Keywords:** Epiphrenic diverticulum, Esophageal diverticulum, Diverticulectomy, Myotomy, Fundoplication

## Abstract

Esophageal epiphrenic diverticulum is a rare condition usually secondary to a primary esophageal motility disorder. Although epiphrenic diverticulum may be treated by thoracoscopic and laparoscopic management, the optimal surgical approach have not been established. We successfully treated a left epiphrenic diverticulum along with achalasia and paraesophageal hernia by a planned combination of thoracoscopic and laparoscopic procedures aided by preoperative simulation using three-dimensional imaging. We reviewed a series of 17 reports on esophageal epiphrenic diverticulum that required either planned or unplanned unexpected transthoracic surgery. The main reasons for requiring a transthoracic approach were adhesions, site and size of the diverticulum, and length of the diverticulum neck. Unplanned procedure changes were required in 12 of the 114 cases for a conversion rate of 10.5%. Diverticulectomy, myotomy, and fundoplication were the most common surgical treatments administered at 42.6%. Based on literature review and our experience, we have developed a flowchart to identify the characteristics of epiphrenic diverticulum cases that require a transthoracic approach. This flowchart can help to determine therapeutic strategies and the optimal surgical approach to esophageal epiphrenic diverticulum treatment and may reduce unplanned changes in the surgery.

## Introduction

Esophageal epiphrenic diverticulum (ED), a pulsion diverticulum occurring in the distal third of the esophagus, is a rare condition with an estimated prevalence of around 0.02–3% [1]. The accepted pathophysiology is that of increased high intraluminal pressure from an outflow obstruction and hypercontractility leading to formation of the diverticulum, and in most cases, esophageal motility disorder is the underlying cause [2]. The main symptoms are dysphagia, regurgitation, weight loss, heartburn, respiratory complaints, and retrosternal pain when swallowing food. Often patients are asymptomatic, but they may suffer from esophagitis, bleeding from ulceration, impaction, and stasis with regurgitation [3–5]. These symptoms either overlap with the symptoms present in achalasia, or other motility disorders are caused by the underlying motility disorder rather than the diverticulum per se, and do not correlate with its size [5–7]. Some argue that an ED may warrant surgery even in asymptomatic patients because of the risk of cancerization of the diverticulum mucosa [8] or spontaneous rupture [9, 10]. When surgical management of the ED is indicated, treatment should focus on the underlying motor disorder and the diverticulum. Three common surgical procedures are diverticulectomy, myotomy, and fundoplication.

The optimal surgical approach for the ED also remains uncertain. The conventional surgical procedure that was usually performed consisted of a thoracotomy [5, 11]. Recent advances in minimally invasive surgery have led to the laparoscopic transhiatal (LT) approach to this disease, and many reports have already been published [9, 10, 12–16]. However, potential disadvantages of the LT approach are the possibility of converting to an unplanned transthoracic (TT) approach if the upper side of the ED is not visible or if there is dense adhesion. Such cases require unexpected changes in positioning or anesthesia technique, e.g., intubation tube exchange for single-lung ventilation would be necessary, and the complication rate due to incomplete diverticulectomy and incomplete myotomy has been reported to be as high as 45% [17]. Therefore, it is very important to determine the optimal surgical approach with sufficient preoperative simulation. We successfully treated ED by a planned combination of thoracoscopic and laparoscopic procedures aided by preoperative simulation using three-dimensional (3D) imaging. We herein describe our surgical technique and approach.

## Case report

**Fig. 1:**
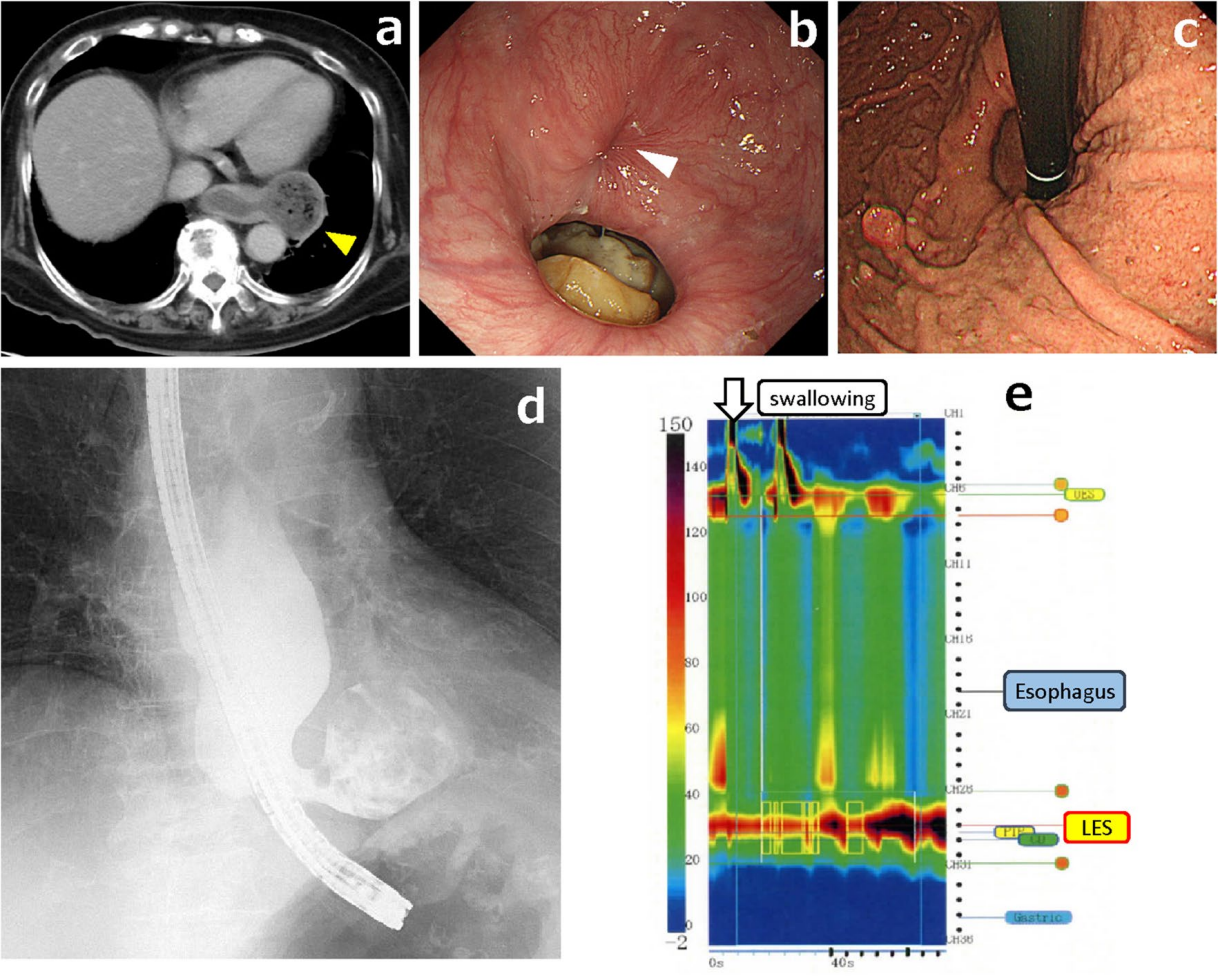
Results of preoperative examination. **a** Computed tomography revealed the epiphrenic diverticulum (ED) in the left thoracic cavity (*yellow arrowhead*).** b** Upper gastrointestinal endoscopy revealed the ED orifice near the esophagogastric junction (*white arrowhead*) and a food residue reservoir. **c** Paraesophageal hernia.** d** Barium esophagram revealed the ED of approximately 7.5 cm in size in the lower thoracic esophagus. **e** Esophageal manometry revealed a median integrated relaxation pressure of 54.6 mmHg, which was indicative of failure of lower esophageal sphincter (LES) relaxation, leading to a diagnosis of achalasia

**Fig. 2:**
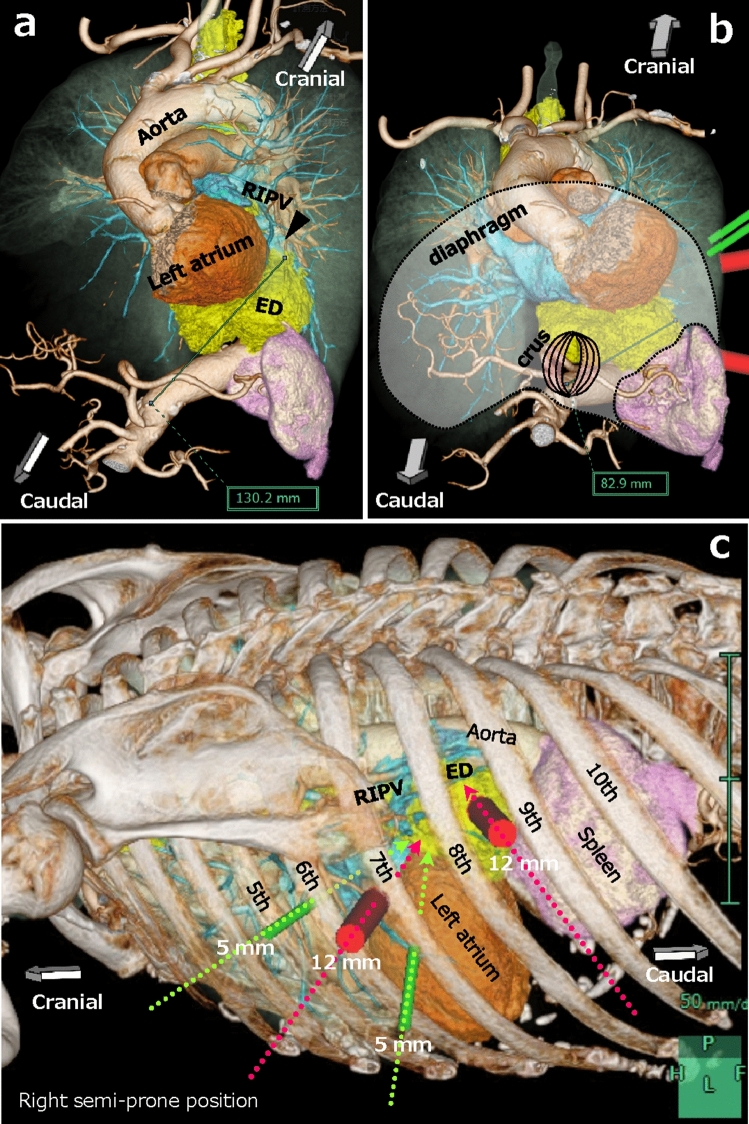
Preoperative simulation using three-dimensional imaging. **a** View from left side of patient. Upper margin of the esophageal epiphrenic diverticulum (ED) reached the level of the right inferior pulmonary vein (RIPV) (*black arrowhead*), 13 cm away. **b** Simulation was also performed in the field of view of the laparoscopic transhiatal approach. The distance from the crus of the diaphragm to the left margin of the diverticulum was 8.3 cm. **c** Preoperative simulation of port placements (12-mm ports in red, 5-mm ports in green) for left video-assisted thoracoscopic surgery in the right semi-prone position

An 80-year-old woman with a body mass index of 31.1 kg/m^2^ presented with chief complaints of postprandial vomiting and weight loss. Subsequent work-up with an esophageal manometry test combined with upper endoscopy, enhanced computed tomography, and barium esophagram confirmed the co-existence of a left ED along with achalasia and paraesophageal hernia (Fig. 1). Although the ED was in the lower thoracic esophagus, 3D images showed that the ED was large (approximately 7.5 cm), and its upper margin reached the level of the right inferior pulmonary vein. The distance from the crus of the diaphragm to the superior margin of the ED was 13 cm and 8.3 cm to the left margin of the ED (Fig. 2a, b). The neck of the ED was relatively small (approximately 2.8 cm), and the orifice was closer to the stomach. However, despite a preoperative fasting period of more than 10 days, much food residue remained inside the ED, and two preoperative endoscopic washes failed to eliminate the contents. We determine the position and number of ports to be used for the left video-assisted thoracoscopic surgery (VATS) for diverticulectomy in the right semi-prone position followed by myotomy and fundoplication via the LT approach by preoperative simulation with 3D images. We chose to use four ports (12 mm at the 6th and 8th and 5 mm at the 5th and 6th intercostal space) in a placement that facilitates access to the diverticulum (Fig. 2c). The 3D image for simulation was created following previously published reports [18, 19].

**Fig. 3:**
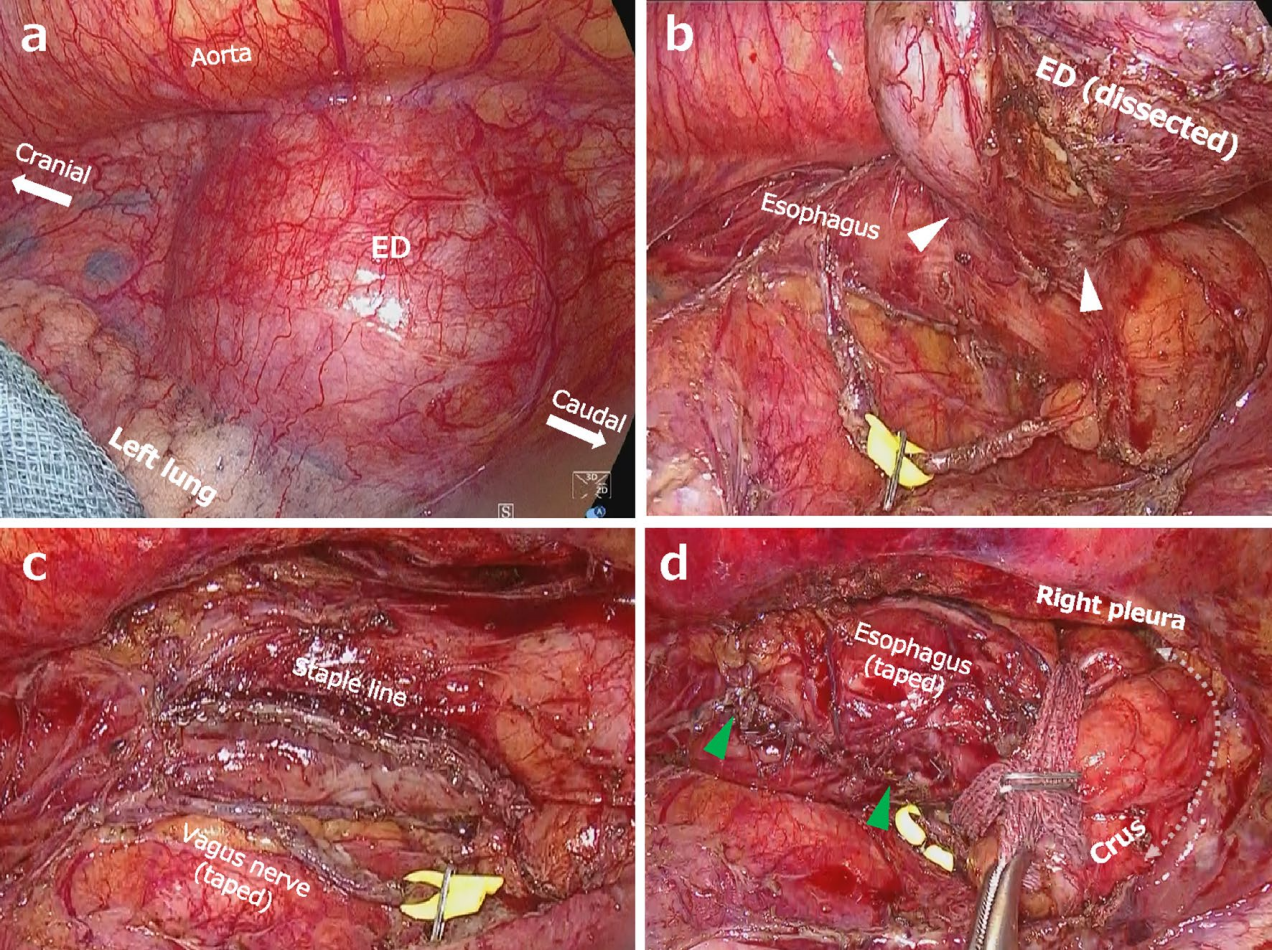
Intraoperative findings. **a** Tight adhesion between the epiphrenic diverticulum (ED) and left lung parenchyma. **b** Exposed neck (*white arrowheads*) of the internally filled ED. **c** After resection of the ED using an endo-stapler. **d** After closing just over the staple line (*green arrowheads*), the phreno-esophageal ligament was dissected circumferentially for the retrograde thoracoscopic transhiatal approach

Tight adherence of the ED to the lung parenchyma required sharp and delicate dissection (Fig. 3a). Intraoperative endoscopic washes also could not expel the food residue from the ED, but the wide operative field of view of the VATS allowed encircling of the ED neck (Fig. 3b). A gastroscope was placed intraoperatively to calibrate the esophagus to prevent stenosis during diverticulectomy, and it was also used to check the integrity of the esophageal wall. The ED was then resected using an endo-stapler (Fig. 3c). The muscular layer was closed over the staple line with a running suture. In preparation for the subsequent the LT approach, which we call the “retrograde thoracoscopic transhiatal approach”, the left crus of the diaphragm was elevated, the phreno-esophageal ligament was dissected and retracted into the thorax to fully expose the esophagogastric junction (EGJ), and then the left vagus nerve and esophagus, respectively, were taped (Fig. 3d).

**Fig. 4:**
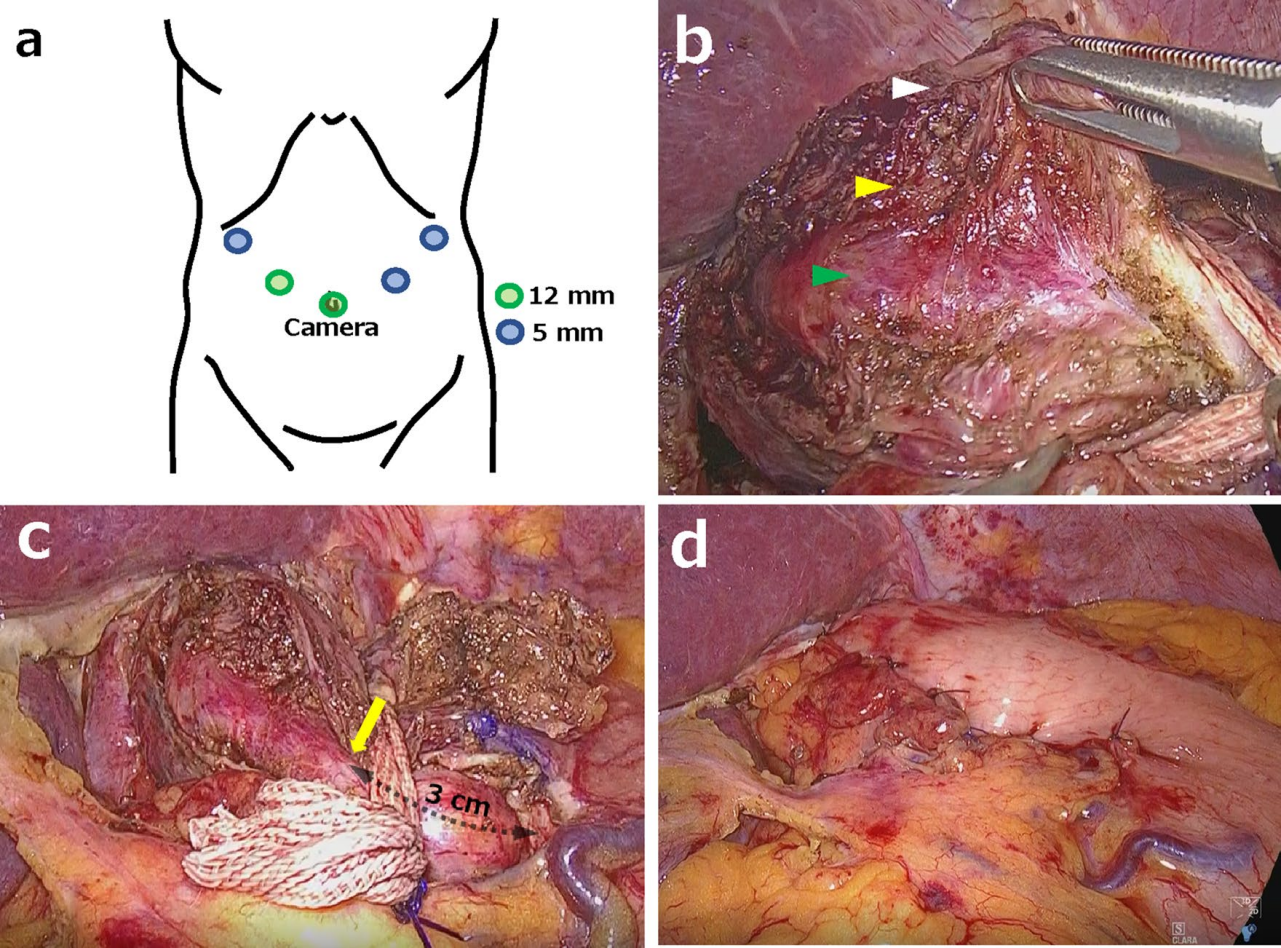
Intraoperative findings. **a** Port placements for the laparoscopic transhiatal approach.** b** A Heller myotomy divides the longitudinal musculature (*white arrowhead*) and circular musculature (*yellow arrowhead*) to expose the mucosa (*green arrowhead*). **c** The myotomy is extended from above the upper limit of the epiphrenic diverticulum neck to almost 3 cm below the esophagogastric junction (*yellow arrow*). **d** After Dor fundoplication

The LT approach in the supine position was performed with 5 ports (Fig. 4a). A Heller myotomy that divides both the longitudinal and circular musculature to reveal the protruding mucosa was performed on the opposite esophageal wall (Fig. 4b). The myotomy was extended from above the upper limit of the ED neck to almost 3 cm below the EGJ (Fig. 4c). After the myotomy was completed, Dor fundoplication was performed to prevent postoperative gastroesophageal reflux disease (Fig. 4d). The resected ED was removed from the abdominal side, which also eliminated the need for a small thoracotomy. The operative time was 116 min (42 min for TT approach and 74 min for LT approach), and the operating blood loss was approximately 10 g. The patient resumed eating on the third postoperative day and was discharged on the fifth postoperative day. After resuming meals, vomiting did not occur even once.

## Discussion

The LT approach has two advantages: first, optimal visualization of both the EGJ and lower mediastinum to perform a long myotomy; and second, perfect alignment of the stapler to the longitudinal axis of the esophagus and the possibility of construction of a fundoplication. With respect to the approach to the ED, Soares et al. reported a significantly shorter median length of stay in the laparoscopic group compared to the thoracotomy group [14]. The LT approach for achalasia has also been shown to result in lower conversion rates, shorter hospital stays, improved dysphagia, and reduced reverse flow compared to the TT approach [20, 21]. However, as in our case, the TT approach is necessary in some cases.

We, therefore, reviewed the results of a series of 17 articles that included esophageal ED cases that require a planned or unplanned TT approach. From 1995 to 2021, a total of 114 patients were treated. We focused on the characteristics of the ED and the reasons necessitating the TT approach in these reports. We also focused on the need for combined thoracoscopic and laparoscopic procedures and thus excluded literature on thoracotomy only and laparoscopic only procedures as well as case reports. The reviewed literature is listed in Table 1, and the clinical data are summarized in Table 2. Of the 114 patients, 102 underwent a planned TT approach and 12 required an unplanned change in approach. There were 17 cases in which VATS and the LT approach were combined in a planned fashion, with no difference with respect to which was performed first. Of the 12 unplanned cases, 8 were converted from VATS to thoracotomy and 2 were converted from the LT approach to thoracotomy, for a conversion rate of about 10.5%. The approach side of the VATS or thoracotomy was described in 101 cases, with 23 performed on the left side and 78 on the right side. Diverticulectomy, myotomy, and fundoplication were the most common surgical treatments administered at 42.6%, followed by diverticulectomy and myotomy at 26.7%.

**Table 1:** Case series that require a planned or unplanned transthoracic approach for ED

Author	Year	*n*	First	Second	R/L	Reason	Treatment*
Peracchia [33]	1995	6	V		R6		D6
		2	V	T (convert)	R2	Adhesion of dive	D2
Saw	1998	1	V			Size	DMF
van der Peet [3]	2001	4	V		R4		DM4
		1	V	LT (convert)	R1	Site, Adhesion of dive	DM
Champion	2003	3	V		L3		DF3
Klaus [29]	2003	1	V	T (convert)	R	Adhesion of dive	DM
Matthews	2003	1	V				DM
Fernando [19]	2005	7	V		R4/L3	Site	DM5, D2
		2	V	LT	R2	Size	DMF2
		1	LT	T (convert)	L	Adhesion of dive	DM
Soares [14]	2011	2	V	T (convert)	L2	Site, Size	DMF2
		2	T			Size, Obesity	DM2
		1	LT	T (convert)	L	Adhesion of dive	DM
Zaninotto [9]	2012	7	LT	T	R7	Site, Size, Large neck	DMF7
Hauge [27]	2014	6	T		L6	Previous surgery	D6
		1	V		L		D
Gonzalez-Calatayud [32]	2014	1	LT	V		Site	DMF
Macke [28]	2015	32	V		R32		DM, D**
		5	V	LT	R5		DMF5
		1	V	T (convert)	R to L	Anomaly of anatomy	DM
		1	V	LP (convert)	R	Intra-abdominal adhesion	DMF
Brandeis	2018	3	T		L3	Site, Large neck	DMF3
		2	LT	V	R2		DMF2
Achim [4]	2017	5	LT	V	R5	Site	DMF5
		2	V		R2	Site	D2
		1	V	T (convert)	R	Intra-abdominal adhesion	DM
Fiorelli	2018	2	V		L2	Site, Size	DMF2
		1	V	LT	R2	Site, Size	DMF
Caso	2019	7	V			Multiple dive 1	DM, D*
		1	V	T (convert)	R	Adhesion of dive	D
		1	LT	V		Previous surgery	DM
Mpaili	2021	1	V		L	Size, Adhesion of dive	DM

*ED* epiphrenic diverticulum, *R* right, *L* left, *V* video-assisted thoracoscopic surgery, *LT* laparoscopic transhiatal approach, *T* thoracotomy, *LP* laparotomy, *D* diverticulectomy, *M* myotomy, *F* fundoplication, *dive* diverticulum

^a^Number indicates number of patients undergoing each procedure

^b^No breakdown provided

**Table 2:** Surgical treatment for the ED used in the 17 published series

Surgical treatment	*n* = 114
Cases of TT approach planned for the ED	102 (89.5%)
VATS	67
Thoracotomy	11
VATS to LT approach	8
LT approach to VATS	9
LT approach to thoracotomy	7
Cases of unexpected TT approach for the ED	12 (10.5%)
VATS convert to thoracotomy	8
LT approach convert to thoracotomy	2
VATS convert to laparotomy	1
VATS convert to LT approach	1
Side of TT approach	
Right	78
Left	23
Unknown	13
Surgical procedure	
Diverticulectomy, myotomy, fundoplication	32 (42.6%)
Diverticulectomy, myotomy	20 (26.7%)
Diverticulectomy, fundoplication	3 (4.0%)
Diverticulectomy	20 (26.7%)
Unknown	39

*ED* epiphrenic diverticulum, *VATS* video-assisted thoracoscopic surgery, *LT* laparoscopic transhiatal

*TT* transthoracic

**Fig. 5:**
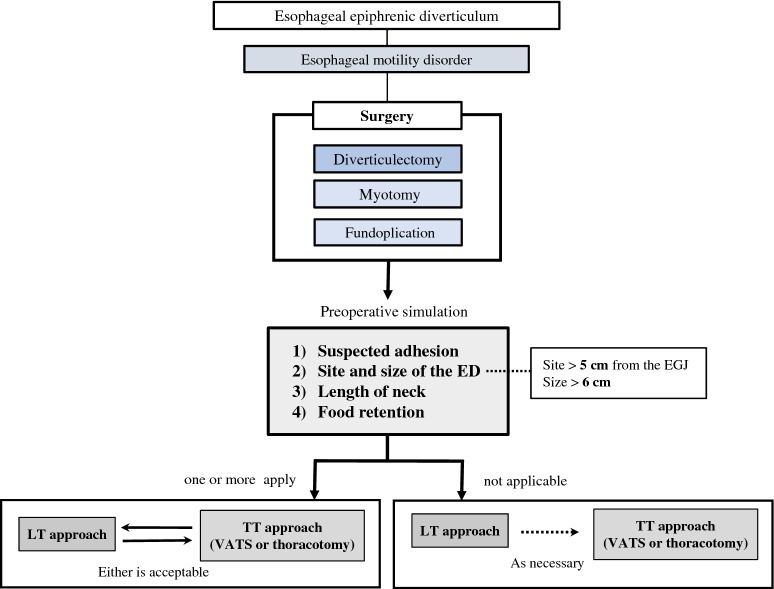
Our flowchart to identify the characteristics of epiphrenic diverticulum cases that require the TT approach based on a literature review. *ED* epiphrenic diverticulum, *EGJ* esophagogastric junction, *LT* laparoscopic transhiatal, *TT* transthoracic, *VATS* video-assisted thoracoscopic surgery

Based on literature review and our experience, we have developed a flowchart to identify the characteristics of epiphrenic diverticulum cases that require a transthoracic approach (Fig. 5). In determining the optimal surgical approach, we believe that the following four items should be thoroughly considered before surgery, based on the results of the literature review and our own experience. First is “Suspected adhesion”. As shown in Table 1, most of the cases that required unexpected surgical conversion were due to diverticular adhesions [3, 14, 19, 23–26]. The TT approach should also be considered if a high degree of intra-abdominal adhesion is expected due to a history of laparotomy [4, 27, 28]. Second is “Site and size of the ED”. Achim et al. reported on a systematic strategy for surgical management of the ED based on the location of the upper border of the ED in relation to the endoscopically identified EGJ [4]. This report recommends a combined VATS and LT approach if the height of the ED neck is more than 5 cm above the EGJ. Many other authors also use 5 cm as the standard [5, 26], Allaix et al. also stated that the inferior border of the ED neck was 56.8 mm or more from EGJ in their study of ED that were difficult to excise with the LT approach [13]. Regarding the size of the diverticulum, literature review indicates that the TT approach may be required when the ED is larger than 6 cm [9, 14, 19, 26, 30, 31]. Third is “Length of neck”. In patients with long ED necks, the entire upper margin of the neck may be difficult to see with the LT approach alone [9, 28]. When the neck is very wide, it may take two or more firings of the endo-stapler to cut it, in which case the points at which suture lines cross over can become a potential site of leakage [9, 13]. Fourth is “Food retention”. As was the case in our patient, even if the neck of the ED is short, poor drainage of internal food residues can also be problematic. The wall of the ED is usually thickened and fibrosed and can be held with a grasper during dissection [26]. However, the narrow lower mediastinal operative field of the LT approach can make it difficult to maneuver the residue-filled ED. If none of these four items is applicable, the case may be possible to complete the LT approach alone, so this should be done first, and then the TT approach can be added if necessary. If one or more items are applicable, the addition of the TT approach may need to be kept in mind, as it may be difficult to safely resect the ED with the LT approach alone.

The flowchart we proposed has limitation. The skill of the physician including anesthesiologist and the device of each institution have a great influence on the decision of which procedure to perform. However, our flowchart, which classified ED cases by risk according to suspected adhesion, site and size of ED, length of the neck, and food retention, may be useful in determining the optimal surgical approach and has the potential to reduce the number of unplanned changes in surgical procedures.

## Abbreviations

ED: Epiphrenic diverticulum
LT: Laparoscopic transhiatal
TT: Transthoracic
3D: Three-dimensional
VATS: Video-assisted thoracoscopic surgery
EGJ: Esophagogastric junction
LES: Lower esophageal sphincter
RIPV: Right inferior pulmonary vein
